# Nurses’ perceptions on implementing a task-shifting/sharing strategy for hypertension management in patients with HIV in Nigeria: a group concept mapping study

**DOI:** 10.1186/s43058-020-00048-y

**Published:** 2020-06-26

**Authors:** Angela Aifah, Deborah Onakomaiya, Juliet Iwelunmor, David Oladele, Titilola Gbajabiamila, Chisom Obiezu-Umeh, Ucheoma Nwaozuru, Adesola Z. Musa, Oliver Ezechi, Gbenga Ogedegbe

**Affiliations:** 1 Department of Population Health, New York University School of Medicine, New York, NY USA; 2 College for Public Health & Social Justice, Saint Louis University, Saint Louis, MO USA; 3 Nigerian Institute of Medical Research, Yaba, Lagos Nigeria

**Keywords:** Nurses’ perceptions, Group concept mapping, Integrated care, Hypertension, HIV care, Task-shifting, Task-sharing

## Abstract

**Background:**

People living with HIV (PWH) in Africa have higher burden of cardiovascular diseases (CVD) compared to the general population, probably due to increased burden of hypertension (HTN). In this study, we explored nurses’ perceptions of factors that may influence the integration of an evidence-based task-shifting/sharing strategy for hypertension control (TASSH) into routine HIV care in Lagos, Nigeria.

**Methods:**

Using group concept mapping, we examined the perceptions of 22 nurses from HIV clinics in Lagos. Participants responded to a focused prompt on the barriers and facilitators of integrating TASSH into HIV care; next, separate focus groups generated relevant statements on these factors; and statements were then sorted and rated on their importance and feasibility of adoption to create cluster maps of related themes. The statements and cluster maps were categorized according to the Consolidated Framework for Implementation Research (CFIR) domains.

**Results:**

All study participants were women and with 2 to 16 years’ experience in the provision of HIV care. From the GCM activities, 81 statements were generated and grouped into 12 themes. The most salient statements reflected the need for ongoing training of HIV nurses in HTN management and challenges in adapting TASSH in HIV clinics. A synthesis of the cluster themes using CFIR showed that most clusters reflected intervention characteristics and inner setting domains. The potential challenges to implementing TASSH included limited hypertension knowledge among HIV nurses and the need for on-going supervision on implementing task-shifting/sharing.

**Conclusions:**

Findings from this study illustrate a variety of opinions regarding the integration of HTN management into HIV care in Nigeria. More importantly, it provides critical, evidence-based support in response to the call to action raised by the 2018 International AIDS Society Conference regarding the need to implement more NCD-HIV integration interventions in low-and middle-income countries through strategies, which enhance human resources. This study provides insight into factors that can facilitate stakeholder engagement in utilizing study results and prioritizing next steps for TASSH integration within HIV care in Nigeria.

Contributions to the literature

- There is an urgent need for evidence-based, integrated care strategies to address the burden of comorbid non-communicable diseases (NCDs) in patients with HIV, which has become more pervasive in low- and middle-income countries (LMICs). Stakeholders’ perceptions are highly important to better adapt these context-specific strategies.
- We found factors related to hypertension training for HIV clinic nurses and on-going supervision to be potential enablers for integrating hypertension management within HIV clinics.
- The findings contribute to the documented gaps in the literature regarding the enablers and barriers of integrating hypertension into HIV care platforms in LMICs.

## Background

Compared to the general population, people living with HIV (PWH) have higher cardiovascular diseases (CVD) related mortality [1, 2], likely due to the increased burden of hypertension (HTN) [3], which affects approximately 14% of PWH in Africa [4]. Based on the Global Burden of Disease report, Nigeria had an increase in mortality from 1990 to 2015 among adults with uncontrolled HTN [5]. Because Nigeria still grapples with a stubbornly unrelenting burden of HIV [6] and a shortage in healthcare workers [7], there is an urgent need for evidence-based strategies targeted at HTN control among PWH, particularly strategies which also address the shortage of healthcare workers.

### Addressing the health worker shortage and HTN control in Nigeria through task-shifting/sharing

Similar to other low- and middle-income countries (LMICs), the acute shortage of healthcare workers is a major barrier to HTN control in Nigeria, which has only 3 physicians per 10,000 population [8]. In particular, this shortage of healthcare workers limits Nigeria’s capacity to control hypertension at the primary care level, where the majority of PWH receive care. To address healthcare human resource challenges as well as the increasing disease burdens within the country, Nigeria’s Ministry of Health (MOH) developed a Task-shifting and Task-sharing Policy for Essential Health Care Services in 2014 with a focus on meeting these challenges at all levels of care [9]. A proven and practical model for improving the management of communicable and non-communicable diseases [10], task-shifting/sharing emphasizes the rational distribution of skills from physicians to underutilized, non-physicians such as nurses and community health workers [7]. While previous studies in Africa have successfully implemented a task-shifting/sharing approach for individual diseases like HIV [11] and hypertension [12], a limited number examine the approach for integrated chronic disease management [13]. Although, the current task-shifting/sharing policy in Nigeria focuses on priority areas such as family and reproductive health, HIV, tuberculosis, and malaria, there is currently no evidence of the policy’s implementation in the *integration of HTN management and control into HIV care*.

### Adapting an evidence-based task-shifting/sharing strategy for hypertension control

In the case of CVD management, task-shifting/sharing has been shown to be an effective strategy in LMIC settings overburdened with healthcare workforce shortages and the increasing prevalence of non-communicable diseases (NCDs) [10]. For example, in a cluster randomized clinical trial of 32 health centers in Ghana, Ogedegbe et al. [7] noted that the addition of a task-shifting strategy for hypertension control or TASSH delivered by trained community health nurses led to a 34% greater reduction in systolic BP than the provision of health insurance coverage alone [7]. Based on the WHO CVD Risk Package [14], components of the TASSH intervention include (1) CVD management clinical decision support using validated WHO risk charts, (2) lifestyle counseling for patients, and (3) medication titration (either increasing a dose or adding a second medication) and patient referral to physicians for the management of complicated HTN [7]. Nurses in the intervention arm were trained in each of these components during yearly training sessions for the study duration [7]. While TASSH has shown to be successful in controlling hypertension in Ghana, the intervention has yet to be applied in integrating hypertension into the HIV care cascade.

### Prioritizing the need to integrate hypertension control into HIV care in Nigeria

With access to highly active antiretroviral treatment (HAART) in LMICs leading to increased survival [2], people living with HIV (PWH) are at an increased risk for NCDs like CVD [15]. The 2018 International AIDS Society Conference acknowledged the growing burden of comorbid NCDs in PWH and proposed an integrated model of care whereby management of NCDs is integrated into HIV chronic care platforms as a cost-effective strategy [15, 16]. Researchers at the meeting also expressed an urgent need for more evidence on implementing integrated HIV-NCD care in LMICs with a critical shortage of health workforce [15]. Based on the successful implementation of TASSH in Ghana [7] along with the similarity in healthcare systems of both countries, the current study explores the perceptions of HIV clinic nurses on the barriers and facilitators of integrating TASSH into routine HIV care for the management of HTN in PWH in Lagos, Nigeria. This information enabled the research team to evaluate the feasibility of implementing TASSH as an integrated model for the management of HTN in HIV clinics for an NIH grant submission, which has since been awarded.

## Methods and data analysis

### Study setting and recruitment

This study took place at the Nigerian Institute of Medical Research (NIMR)—a research arm of the Federal Ministry of Health, Nigeria. NIMR offers a wide spectrum of public health services and clinical research in HIV, tuberculosis, malaria, neglected tropical diseases, maternal and reproductive health, and NCDs among others. It has a comprehensive HIV treatment center with over 25,000 PWH cumulatively enrolled in care since 2004. A total of 22 HIV clinic nurses were recruited, from 20 HIV clinics in Lagos, to participate in the study through convenience sampling wherein supervisors selected nurses based on staffing availability. The 20 HIV clinics were a subset of the clinics that will be selected for the proposed intervention. In addition to being representative of the 20 HIV clinics, all of the HIV nurses participated in the brainstorming as well as the sorting and rating activities while 18 of the 22 nurses participated in the group concept mapping (GCM) discussion activity. The rationale for the sample size was based on similar studies using GCM strategy [17]. All participants provided written informed consent and the study was approved by NIMR’s Institutional Review Board.

### Study objective and design

**Fig. 1:**
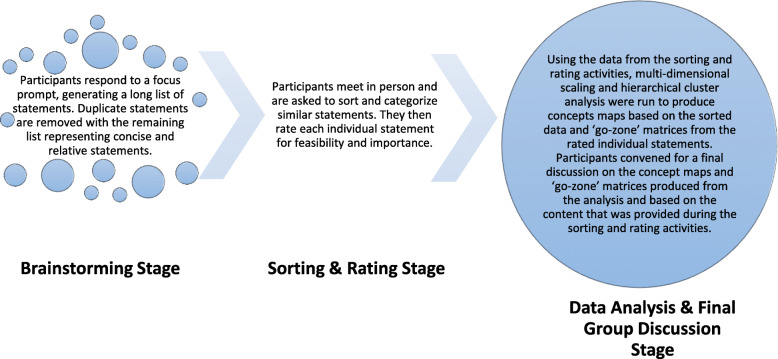
GCM stages

To better understand HIV nurses’ perceptions on TASSH, we employed GCM methodology. GCM uses a mixed-methods participatory approach to stakeholder engagement, in a six-step process (preparation, generation, structuring, representation, interpretation, and utilization) to assess the perceptions of participants on a specific topic by generating illustrative conceptual frameworks of the target group’s views [18, 19]. For this study, HIV clinic nurses were engaged in GCM activities to elicit their thoughts on an integrated HIV-NCD model using a task-shifting/sharing strategy for the management of hypertension in PWH. GCM analyses were conducted using the Concept Systems Global Max software (Concept Systems, Inc., Ithaca, NY) [18]. Figure 1 depicts how the GCM methodology was adapted for the purposes of this study in the following stages: brainstorming, sorting and rating, and GCM data analysis and final group discussion. At the first GCM group meeting, a discussion took place on the aims and outcomes of the Ghana TASSH intervention study to address any questions participants had about the intervention. Data was collected in March 2019.

### Brainstorming stage

The nurses provided succinct statements in response to the following prompt: “*In order for HIV clinic nurses to successfully adopt a task-shifting/sharing strategy for hypertension control among people living with HIV, they need*....” Responses were manually entered into the Concept Systems software to generate a list of statements that reflects each nurse’s response by creating individual profiles for each nurse with a unique ID code. This process yielded a final set of statements which were randomly sorted and printed out on separate sorting cards for group discussion. Nurses also responded to a brief demographic survey on their applicable HIV work experience.

### Sorting and rating stage

The nurses participated in two separate focus group sessions [led by AA and DO], where they worked in pairs to sort the final list of statements into grouped piles that made sense to them and based on their current practice within the HIV clinics. Participants were instructed to create as many piles as needed while making sure there were not too many or too few piles. After sorting the statements, they then rated each statement on their perceived importance [on a 3-point scale, with 1 being “important” and 3 being “very important”] and potential feasibility [on a 4-point Likert scale, with 1 being “not feasible” and 4 being “extremely feasible”] concerning the proposed intervention [TASSH].

## Data analysis

After sorting and rating, the final statements were entered into the Concept Mapping software, which uses multidimensional scaling (MDS) analysis to graphically show points representing statements that were frequently sorted together being more proximal to each other and infrequently sorted statements being further apart [20]. MDS produces several visual outputs including a point map, cluster map, and go-zone rating—all of which are shared with the participants to discuss their interpretability and to come to an agreement on the representativeness of the raw data with the outputs [19, 21]. For example, the “cluster map” output shows how the statements generated, represented by points, were grouped by participants [21]. In this study, the MDS yielded two viable cluster maps representing 8 and 12 cluster groupings of statements.

### Interpreting the GCM maps through final group discussion

Following the MDS analysis, another focus group with a subset of the nurses (18 total) was convened to provide them feedback on the two cluster maps and the final cluster map groupings and discuss their views. For this purpose, we printed out the cluster maps and shared the analytical outputs with the nurses. The nurses were asked to vote on which cluster, i.e., choosing between the 8 or 12 cluster grouping, was most representative of their perspectives of TASSH. They were also asked to provide feedback on potential adaptations to TASSH based on their interpretation of the cluster maps. The nurses were also presented with Go-Zone matrices which show sets of rated statements in the cluster maps, where each statement is assigned coordinates based on mean rating on *x*- and *y*-axis. The Go-Zone creates four quadrants based on ratings of the statements (above or below the mean) for its importance and feasibility of integrating it into the proposed intervention [TASSH]. The Go-Zone creates the following zones: high importance-high feasibility (green zone), high importance-low feasibility (yellow zone), low importance-high feasibility (orange zone), and low importance-low feasibility (gray zone). The high-high quadrant (i.e., high importance and high feasibility) is typically considered the Go-Zone because it includes the ideas rated most highly for both criteria, which in this case, is the importance and feasibility of the rated statement for integrating hypertension management into HIV care via a task-shifting/sharing strategy. At the conclusion of this focus group, the nurses also provided additional feedback on specific challenges for integrating an intervention like TASSH into HIV care. Inductive thematic analysis was conducted for this final feedback.

### Mapping the clustered themes to CFIR

Following the group GCM activities, we used the Consolidated Framework of Implementation Research (CFIR) to map the 12 clusters and further assess how they could be tailored for the implementation of the intervention. CFIR also provides a practical guide for evaluating stakeholders’ perceptions on the feasibility and importance of implementing an evidence-based program, in this case, TASSH. Developed by Damschroder and colleagues, CFIR provides an overarching typology for the systematic assessment of factors, which may influence an intervention’s implementation and effectiveness [22, 23]. Consisting of five domains (intervention characteristics, outer setting, inner setting, characteristics of the individuals involved, and the process of implementation), each with their related constructs, CFIR has been used as a rapid-cycle evaluation guide of stakeholder’s perceptions on the implementation of a healthcare intervention [23].

## Results

**Fig. 2:**
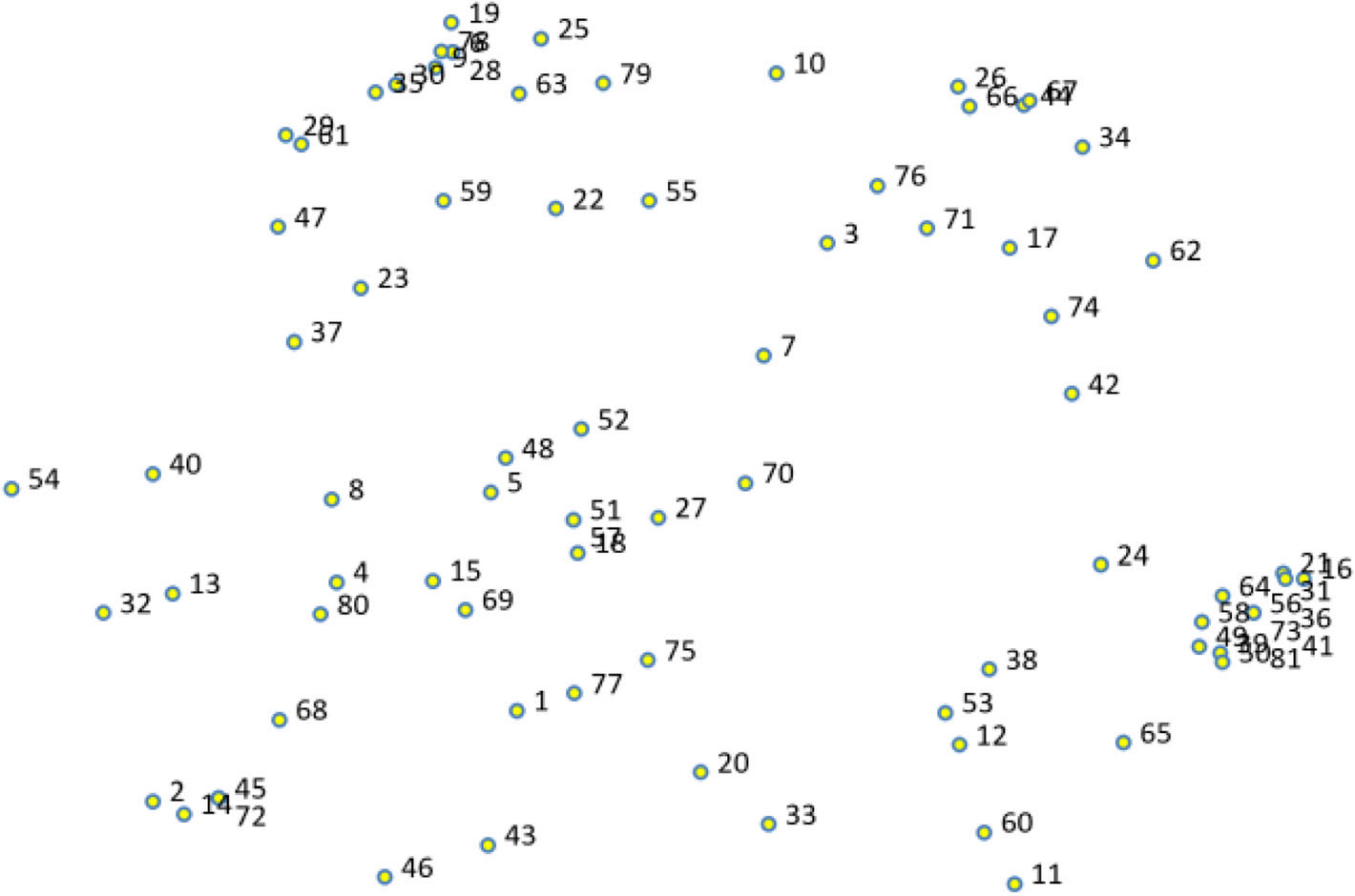
Final point map

Applying GCM, nurses generated a total of 125 statements, which were reviewed by two members of the research team (AA and DO) to remove redundant statements and statements not relevant to the prompt, leaving a final set of 81 statements. Figure 2 represents the point map of the 81 statements, depicting the varied spread and relative closeness of the statements. In the following sections, we provide the findings on participant’s TASSH-related demographic data, results from the GCM activities, the mapping of the 12 clusters to the CFIR domains, and feedback on specific challenges for integrating TASSH.

### Participants’ TASSH-related demographic data

All study participants were women, with 2 to 16 years’ experience in the provision of HIV care. Initial group discussions with participants showed that they were familiar with the task-shifting/sharing approach but had not participated in a task-shifting/sharing intervention for integrating hypertension management and control into HIV treatment at their local clinics. The nurses had worked in HIV care for an average of 6 years, and they care for approximately 79 patients per day, of which about 41 are PWH. Regarding the essential duties of the nurses specifically related to components of the proposed TASSH intervention, 60% routinely perform finger prick glucose test; 67% conduct urine dipstick, with approximately 94% engaged in patient education and counseling on lifestyle modification.

### GCM activities’ output

**Fig. 3:**
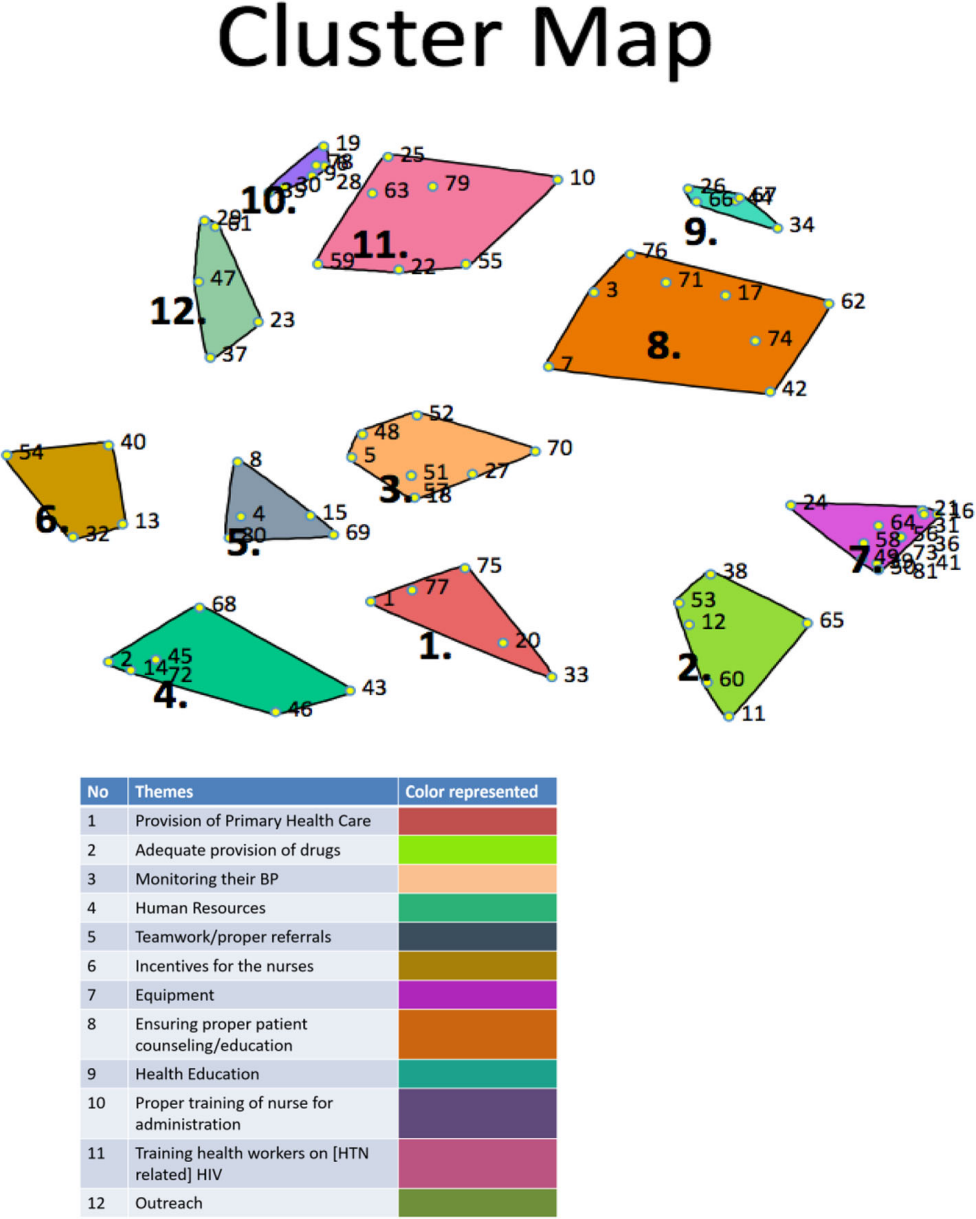
Cluster map with corresponding legend for the 12 themes

As noted in Fig. 3, the nurses selected a 12-cluster map which they believed best characterized their understanding of the key factors that may facilitate or hinder the integration of hypertension management into HIV care using a task-shifting/sharing strategy. For GCM, and through the MDS analysis, a “stress value” of the point map is typically generated to determine how well the MDS solution maps to the inputted data and indicates a goodness of fit [21]. In this study, the stress value for the cluster maps was 0.29 demonstrating overall good fit. The representative statements for each of the 12 clusters along with their corresponding number are shown in Table 1.

**Table 1:** Twelve clusters with corresponding statements

Themes	Statements
1. Provision of primary health care	1. Their closeness to the facility is considered too.
	20. Availability of drugs at an affordable price for patient consumption.
	33. Restructuring the PHC setting in such a way that the nurse is officially allowed to see the sick when the doctor is not around or very busy especially at the outpatient clinic and emergency clinic.
	75. Making ART clinic a one-stop clinic for PLHIV/hypertension to reduce waiting time.
	77. Prompt linkage of hypertensive clients to physician and other relevant facilities for further management.
2. Adequate provision of drugs	11. Financial support.
	12. Provision of primary health care.
	38. Working environment should be more conducive for the health worker clients.
	53. Conducive work environment.
	60. Provision of subsidized anti-hypertensive drugs.
	65. Drugs for management of hypertension should be available in the HIV clinic.
3. Monitoring their BP	5. Ensure regular monitoring of BP at every clinic visit.
	18. Monitor vital sign regularly.
	27. Proper clinic standard operating procedure (SOP).
	48. Proper follow-up strategies on clients.
	51. Monitor intake and output chart.
	52. Great nurse/patients relationship
	57. Monitoring of blood pressure.
	70. Proper supervision of standard operating procedure (SOP).
4. Human resources	2. Employ more nurses.
	14. Availability of human resources.
	43. The PHC organogram should be revisited to put the nurse in their proper position.
	45. Human resources—more nurses.
	46. More nurses to be employed into the PHC setting.
	68. Relegation of power—let people take responsibility.
	72. Adequate man power.
5. Teamwork/proper referrals	4. Ensuring that each clinic, i.e., both HIV and medical outpatient clinic (hypertension) falls on the same visit.
	8. Supervision of staff on providing care.
	15. Aligning both clinic appointments to fall on the same day.
	69. Providing measures in improving their health care, i.e., the clinic should be interwoven (the appointment should always be the same date) to avoid excessive stress.
	80. Effective teamwork.
6. Incentives for the nurses	13. Encouragement of work delegation.
	32. Consideration of the number of nurses in a clinic
	40. There should be clarity on which task is shifting to who, to avoid unnecessary rivalry among health workers
	54. Incentive for the nurses.
7. Equipment	16. More working tools like protective measure—gloves, apron, boots, cap and face mask, and testing kits.
	21. Provision of blood pressure apparatus.
	24. Blood pressure monitoring manual.
	31. Provision of functioning sphygmomanometer at the clinic always.
	36. Adequate provision of materials/equipment.
	39. Government should be ready to supply and provide everything needed for
	41. We need materials or instrument to manage the HIV/hypertension patient.
	49. Provision of HIV test kit and drugs.
	50. Provision of facilities needed.
	56. Available equipment to work with.
	58. Weighting scales (SECA) with height.
	64. Enhance current medical equipment
	73. Regular supply of working tools and necessary materials.
	81. Provision of better equipment to work with.
8. Ensuring proper patient counseling/education	3. Counseling of patient for follow-up.
	7. Proper follow-up and non-abrupt stoppage of BP drug.
	17. Provision of counseling to the patients.
	42. Ensuring that each patient has their BP apparatus to monitor their blood.
	62. Ensuring daily use of anti-hypertensive drug.
	71. Proper health education on diet and lifestyle.
	74. Encourage clients to have personal BP apparatus for their home use, in order to identify BP and report before appointment day if necessary.
	76. Proper health education on hypertension with people living with HIV during each visit to the clinic.
9. Health education	26. Re-educating patients on importance of diet, rest and adherence in the management of HIV/hypertension.
	34. Encouraging patient on the importance of adherence to drug regimen to prevent relapse in healthcare.
	44. Stressing the do’s and don’ts of people living with HIV and hypertension, i.e., smoking, drinking of alcohol, and use of protective materials during sexual intercourse.
	66. We need to give them quality counseling.
	67. Intensified health education on hypertension with people living with HIV on clinic days.
10. Proper training of nurses for administration	6. Training for nurse to improve their knowledge on hypertension.
	9. Nurses need better understanding of the case.
	19. Proper training of nurses for administration.
	28. Training to nurses on how to provide psycho-social support to patients.
	30. Training of health workers on the update of HIV drugs.
	35. Constant continuous communication in the form of training and re- training for nurses.
	78. We need proper training to be able to manage the patients.
11. Training for health workers on [HTN related] HIV	10. Personal training on adherence counseling on lifestyle modification, medication, and diet.
	22. We need to know the patient's problems.
	25. Training on screening patient with cardiovascular risk.
	55. Provide detailed and good understanding of task-shifting strategies for hypertension control.
	59. Constant review of training workshop of nurses and more health workers on ground.
	63. Adequate knowledge of anti-retrovirals that can affect blood pressure.
	79. Knowledge on the treatment of hypertension.
12. Outreach	23. Early detection of people living with hypertension.
	29. Sensitization.
	37. Prioritizing patient health care according to their health care needs.
	47. There should be good advocacy, communication between health workers and the community to know prevention and precaution and how to go about it.
	61. Awareness—sensitize the environment.

**Fig. 4:**
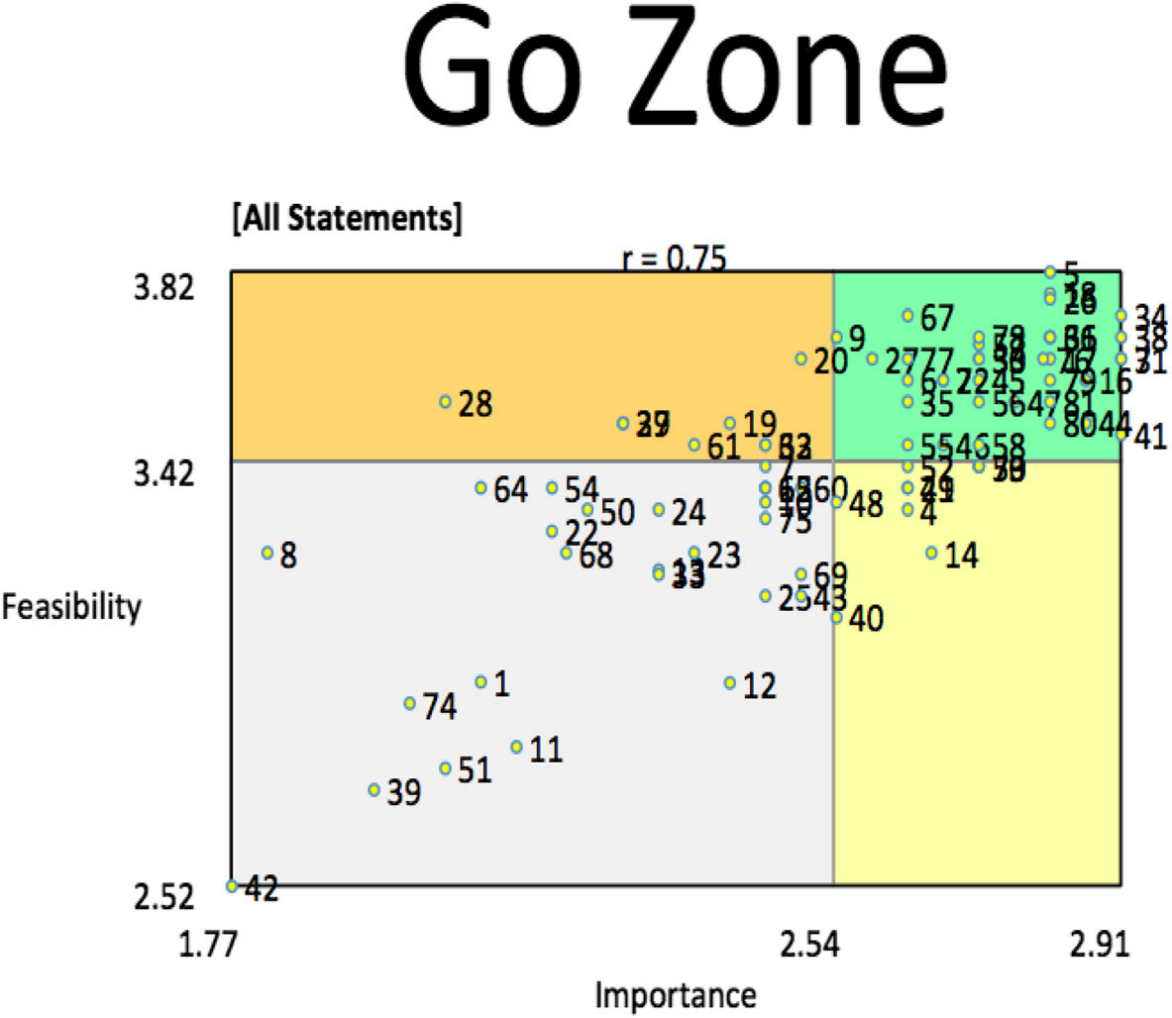
Feasibility and importance Go-Zone

Figure 4 shows the Go-Zone ratings generated for all 81 statements and the 12 cluster map. Five statements were rated high for importance and feasibility of integrating hypertension management into HIV care. These include “Ensure regular monitoring of BP at every clinic visit” (statement 5); “Provision of counselling to the patients” (statement 17); “Monitor vital signs regularly” (statement 18); “Re-educating patients on importance of diet, rest, and adherence in the management of HIV/Hypertension” (statement 26); and “Encouraging patient on the importance of adherence to drug regimen to prevent relapse in healthcare” (statement 34). Conversely, the statement rated the lowest for importance and feasibility was “Ensuring that each patient has their BP apparatus to monitor their blood pressure at regular intervals and comes to the hospital in case there is any rise in blood pressure” (statement 42).

### The 12 cluster themes and the CFIR domains

Based on the discussions with the nurses and their applicability to the study, we mapped the themes or clusters to four domains of CFIR—i.e., inner settings, outer settings, intervention characteristics, and characteristics of the individuals involved. The fifth domain, implementation process, was not applicable for the purpose of this study. Table 2 shows the CFIR domains and the corresponding theme or cluster. The majority of the 12 cluster themes selected by nurses were considered by the research team to be most applicable to the *inner setting* and *intervention characteristics* domains. While the *individuals involved* domain only had one cluster theme that seemed most relevant.

**Table 2:** CFIR domains and the associated clusters

CFIR domains	Associated cluster themes
Inner setting: features of the healthcare system within which TASSH is implemented	• Human resources • Teamwork/proper referrals • Equipment • Outreach
Outer setting: the external context to the settings where TASSH is being implemented	• Incentives for nurses • Provision of primary health care access • Adequate provision of drugs
Intervention characteristics: features of the proposed intervention	• Training health workers on management of [HTN related] HIV • Ensuring proper patient counseling/education • Health education • Proper training of nurses on components of the intervention
Individuals involved: characteristics of participants involved in the implementation	• Monitoring their [patient] BP

### Feedback on specific challenges for integration

After the GCM discussions and as part of their closing remarks at the final group meeting, the nurses provided additional feedback on content specific barriers that might hinder the integration of hypertension management into HIV care in their clinics. The barriers identified were divided into the following categories: nurses’ perspectives on work-related challenges and nurses’ perspectives on patient barriers. Nurses’ perspectives on work-related challenges included the shortage of nurses at primary healthcare centers; work ethics of health workers (including nurses); limited knowledge of hypertension, CVD risk assessment, and prescribing hypertension medication; and inadequate provision of tools/ resources for hypertension management. Regarding nurses’ perspectives on patient barriers, the following were highlighted: patients’ limited knowledge of hypertension and self-management; the inability of patients to prioritize payments for anti-hypertensive medication in addition to HIV medication; and high medication cost.

## Discussion

To our knowledge, this is the first study to use a rigorous mixed-methods GCM methodology to explore the perceptions of HIV clinic nurses on the factors that may facilitate or hinder the integration of hypertension management into the HIV care using an evidence-based task-shifting/sharing strategy for hypertension control. We identified 81 statements, which were grouped into 12 clusters representing key factors HIV nurses chose as most comprehensive or fitting for implementing a task-shifting/sharing strategy to integrate hypertension management into HIV care.

As noted in the cluster map and based on other studies using concept mapping [24], the points or statements which are closer denote those themes which the MDS analysis found to be more closely related than those points which are farther apart. The same logic applies to the clusters themselves, i.e., those in close proximity are conceptually related in comparison to those farther apart. In terms of the size or shape of the clusters, Schell and Luke [24] note that typically smaller, denser clusters represent concepts or statements which the HIV nurses believed to be more acceptable or compatible; while the larger clusters denote more dispersed ideas. Interestingly, statements represented in clusters 7 (“equipment”), 10 (“proper training of nurses for administration”), and 9 (“health Education”) were proximal to one another, showing that the statements for each cluster were closely related to each other. These three clusters were also among the smaller, denser groupings—meaning that HIV nurses found these statements to be more acceptable for implementing a task-shifting/sharing strategy for hypertension management. Additionally, the stress value of 0.29 indicates that the final cluster was representative of the nurses’ perspectives. For GCM, a lower stress value indicates a better fit and reflects a stronger relationship between optimal and actual configurations [21]. Other studies have used a value of 0.35 or greater to indicate a higher stress value, i.e., a greater discrepancy between the inputted data and the generated concept map [25]. Our findings are in line with those of other studies that evaluated task-shifting/sharing strategy for hypertension management [7, 12, 17, 26]. Blackstone et al. [26] also noted that proper training of nurses was particularly important for sustaining a task-shifting/sharing strategy for hypertension management.

This study extends our previous work on the capabilities, opportunities, and motivations of HIV clinics in Lagos State’s practice capacity to integrate hypertension management into HIV care [27], by specifically focusing on the perspectives of the HIV clinic nurses on the barriers and facilitators of implementing a task-shifting/ sharing intervention. The application of GCM noted that for HIV nurses, implementing TASSH as part of the routine care was not only feasible but also regarded as highly important to adequately address the burden of hypertension among PWH. Furthermore, the low stress value from the concept mapping analysis shows congruency in the sorted and rated statements by the HIV nurses and the generated maps -  validating that this mixed-methods approach adequately represented the perceptions of the HIV nurses. Other GCM studies have also noted similar findings regarding the representativeness of a lower stress value for the final model with the statements generated by participants [26, 28].

Findings from our study underscore the importance of developing nurse-based strategies to integrate hypertension management into HIV care given the limited availability of evidence supporting context-specific strategies in Africa [29]. A recent review by Vorkoper and colleagues noted that implementation science research will be critical in efforts to integrate evidence-based interventions into existing HIV chronic care platforms given the emphasis on context-appropriate approaches for sustained health interventions [29]. One such example of a flexible, adaptable implementation science framework is CFIR. The application of CFIR to the 12 clusters or themes chosen by the nurses is a useful approach for evaluating which factors or domains are most relevant for an intervention that is being tailored to a specific context. While all five CFIR domains are most commonly used to understand the context within which evidence-based strategies are implemented [23], in our study, the domain(s) which stood out the most, i.e., those associated with the majority of the themes were *inner settings* and *intervention characteristics*, with each matched to 4 of the 12 themes. The fact that most clusters were more applicable to a few CFIR domains as opposed to all, points to the need to consider the specific contextual factors which may have more implications on the implementation and/or adaptation of an intervention like TASSH in a different setting, particularly for integrated care platforms. Although we are not aware if other studies have applied CFIR when analyzing stakeholder’s perceptions of task-shifting/sharing strategy for hypertension management in PWH in LMICs, our findings underscore the applicability of using CFIR as an analytical guide for implementing and adapting stakeholder’s perceptions of evidence-based interventions.

## Limitations and strengths

There were a few limitations. First, we only sampled 22 nurses, most of whom were staff nurses, but did not include other allied health workers [e.g., Community Health Extension Workers] who might also provide care in HIV clinics in Nigeria. Such a small sample size along with the lack of variety in HIV clinic health workers limits the generalizability of the study findings. Despite this limitation, an asset of the study is the inclusion of participants from HIV clinics where the proposed intervention will be implemented. Second, the concept mapping exercise was labor intensive and required several hours of focus and participation by the nurses over the course of 2 days. This might have prevented more nurses from participating because of work demands at their various health facilities. Third, not all the nurses from the first session completed the concept mapping exercise from the second session (though all nurses at the concept mapping session were part of the first session). While it would have been ideal that all of the HIV clinic nurses participated in all of the GCM related activities and sessions, an advantage of GCM is that it is not a requirement to have every single person participate in each activity [30]. Finally, the mapping of the CFIR domains to the 12 cluster themes did not follow a specific analytical approach but was rather based on a simple synthesis of the themes based on the feedback from the HIV nurses. A more thorough content analysis using qualitative methods may have provided additional insight on the applicability of CFIR to the 12 clusters. Despite these limitations, the use of concept mapping supports the application of novel, mixed methods approaches for engaging stakeholders in LMICs who are primarily responsible for adapting task-shifting/sharing strategies.

## Implications

Given the increasing need to address NCDs among PWH in regions with shortages in healthcare workers, interventions tailored to address the dual disease burden while simultaneously enhancing the capacity of key healthcare professionals are of most importance. Despite the establishment of a task-shifting/sharing policy and the prevalence of hypertension among PWHs, Nigeria has yet to institute the approach for integrating NCDs like hypertension into the HIV care continuum. The current study provides contextual evidence of potential barriers and facilitators for implementing a proven task-shifting/sharing intervention for hypertension management within the Lagos State primary healthcare system. Notably, the findings point to factors such as additional health education, human resources, training nurses on hypertension-related HIV treatment, and proper patient counseling or education, which may influence the implementation of a task-shifting/sharing intervention for hypertension management. For an intervention like TASSH to be successfully adopted as part of the routine care offered, there must be targeted consideration for incorporating these factors for integrating hypertension care while simultaneously addressing healthcare worker shortages in HIV clinics in Lagos, Nigeria. Given the burden of comorbid HIV and NCDs in LMICs, future research should consider the feasibility of scaling up task-shifting/sharing strategy for integrating hypertension management into the HIV care cascade across impacted countries and regions.

## Conclusion

Engaging and understanding stakeholders’ perceptions of feasibility of an evidence-based intervention beyond its initial implementation, can provide critical insights for developing sustainable programs in LMICs [17]. Nurses play a major role in the implementation of task-shifting strategies, particularly when addressing integrated care platforms for comorbid HIV and hypertension. For these reasons and more, this study provides critical, evidence-based support in response to the call to action raised by the 2018 International AIDS Society Conference regarding the need to implement more NCD-HIV integration interventions in LMICs through strategies that enhance human resources. As non-communicable diseases become more pervasive among PWH in LMICs still overburdened with communicable diseases and shortages in healthcare workers, context-specific integrated care platforms will be critical in addressing dual disease burden, particularly among PWH and comorbid NCDs. Findings from our study provide highly relevant and emergent evidence in support of the exploration of key stakeholders’ perceptions on the feasibility and importance of implementing a task-shifting/sharing strategy for the integration of hypertension management into HIV care.

## Data Availability

All data generated or analyzed during this study are included in this published article.

## Abbreviations

NIMR: Nigerian Institute of Medical Research
HAART: Highly active antiretroviral therapy
GCM: Group concept mapping
WHO: World Health Organization
CFIR: Consolidated Framework for Implementation Research
CVD: Cardiovascular disease
MOH: Ministry of Health
RCT: Randomized controlled trial
HTN: Hypertension
TASSH: Task-shifting strategy for hypertension control
HIV: Human immunodeficiency viruses
AIDS: Acquired immunodeficiency syndrome
PWH: People living with HIV
NCDs: Non-communicable diseases
LMICs: Low- and middle-income countries
AA: Angela Aifah
DO: Deborah Onakomaiya
JI: Juliet Iwelunmor
DAO: David Oladele
CO: Chisom Obiezu-Umeh
UN: Ucheoma Nwaozuru
TG: Titilola Gbajabiamila
AM: Adesola Z. Musa
OE: Oliver Ezechi
GO: Gbenga Ogedegbe
